# Effect of Soil Organic Matters in Dredged Soils to Utilization of their Mixtures Made with a Steel Slag

**DOI:** 10.3390/ma13235450

**Published:** 2020-11-30

**Authors:** Kanako Toda, Ryosuke Kikuchi, Tsubasa Otake, Satoshi Nishimura, Yuzoh Akashi, Michihiro Aimoto, Takeshi Kokado, Tsutomu Sato

**Affiliations:** 1 Division of Sustainable Resources Engineering, Faculty of Engineering, Hokkaido University, N13W8, Kita-ku, Sapporo, Hokkaido 060-8628, Japan; rkikuchi@eng.hokudai.ac.jp (R.K.); totake@eng.hokudai.ac.jp (T.O.); tomsato@eng.hokudai.ac.jp (T.S.); 2 Division of Field Engineering for the Environment, Faculty of Engineering, Hokkaido University, N13W8, Kita-ku, Sapporo, Hokkaido 060-8628, Japan; nishimura@eng.hokudai.ac.jp; 3 Nippon Steel Corporation, Marunouchi 2-6-1, Chiyoda-ku, Tokyo 100-8071, Japan; akashi.n9t.yuzoh@jp.nipponsteel.com; 4 Advanced Technology Research Laboratories, Nippon Steel Corporation, 20-1 Shintomi, Futtsu-shi, Chiba 293-8511, Japan; aimoto.d7k.michihiro@jp.nipponsteel.com; 5 Nippon Steel Eco-Tech Corporation, Kyobashi 1-18-1, Chuo-ku, Tokyo 104-0031, Japan; kokado.takeshi.29x@eco-tech.nipponsteel.com

**Keywords:** steel slag, dredged soil, soil organic matters, strength inhibition, pozzolanic reaction

## Abstract

Dredged soils have been used as construction materials by alkaline activation with steel slag (steel slag-dredged soil mixtures) at harbors. Such mixtures develop strength chiefly by calcium silicate hydrate (C-S-H) formation by the pozzolanic reaction. However, the strength of such mixtures is unpredictable, and in some cases, mixtures have been too soft for the intended engineering application. An identification of strength development indicators would accelerate evaluation processes for strength development to facilitate and promote the utilization of such materials. This paper focuses on the relationship between the characteristics of soil organic matters in dredged soils and the strength development of the mixtures by a comparison of eight dredged soils collected from eight different Japanese harbors. The characteristics of the soil organic matters were identified to determine as indicators of mixtures with weak strength development, i.e., enriched sulfur content in extracted soil organic matter (humic acid) fraction, and the N/C ratio of humic acid similar to land humic acid standards. Increases in the validated fraction of dredged soils and steel slag by replacing fractions disadvantageous to construction resources would contribute to reduce waste production, which would lower the environmental impact of the use, aiming to achieve sustainable utilization of such materials.

## 1. Introduction

The excavation of soil sediments in water ways for shipping safety produces significant volumes of dredged soils (e.g., [1,2,3]). In Japan, 16 million tons of marine soils were dredged in 2013 and used landfill (59%), made beach reclamation and sand covers (25%), or disposed in the ocean (5%) along with other minor options for use and disposal [4]. However, limitations on landfill sites and the environmental impact of its creation, which consumes land and other primary resources for site construction such as cement and aggregate, has highlighted the necessity of dredged soil utilization improvements.

The concretion of dredged soils by mixing alkaline activators [5,6,7,8,9] makes them useful as construction materials and offers the potential to promote their utilization. Furthermore, parts of the dredged soils have also been mixed with industrial waste such as fly ash, lime production waste, and steel slag. Steel slag is a by-product in the iron-making process, and it has the ability to develop the strength with dredged soils by mixing and curing at room temperature [10,11,12]. It is a kind of alkaline activator of dredged soils. Steel slag-dredged soil mixtures are utilized in engineering works close to the seashore because of the ease of material transport and application.

However, some steel slag-dredged soil mixtures exhibit weak strength in their construction. In addition, the final strength of the mixtures is unpredictable before mixing, which detracts from usefulness in the utilization of the mixtures. Currently, the utilization of mixtures require numerous strength tests with trial and error before the application. Unpredictable strength development especially results from the components of dredged soils where their effect on hardening is not fully elucidated. Such soils are composed of fine minerals, clay minerals, inorganic amorphous phases, and soil organic matters. The strength development of steel slag-dredged soil mixtures is presumably attributed to the extent of calcium silicate hydrate (C-S-H) phase formation by the pozzolanic reaction [10,13]. Pozzolanic reaction refers to the formation of C-S-H from portlandite (Ca(OH)_2_) and siliceous materials in moist conditions, where C-S-H is known to act as a major binder in calcium-based cementitious materials [14]. Therefore, a total clarification of key components that affect the pozzolanic reaction, the strength development of the steel slag-dredged soil mixtures, may overcome the problem as it would make evaluation processes faster for the strength development of mixtures that should facilitate their utilization.

In order to evaluate the factors affecting the strength development of steel slag-dredged soil mixtures, previous studies investigated four dredged soils obtained from different Japanese harbors and their mixtures with two steel slags [13]. These studies showed that the inorganic amorphous silica content of dredged soils, comprising diatom frustules and volcanic glasses, is a key factor affecting the extent of silica supply to form C-S-H. Furthermore, as discussed in Kiso et al. [10], portlandite content in steel slag is confirmed as another key factor, which is a major supplier of dissolved calcium and achieves alkaline conditions, which enables the dissolution of silicate phases and the precipitation of C-S-H.

However, some mixtures made with the dredged soils and steel slag with sufficient amorphous silica and portlandite showed no strength development in unconfined compressive strength tests. This potentially indicated the existence of other factors that affect the strength development of these mixtures. Some organic matter including humic acid, an extractable fraction of soil organic matters, are known to inhibit the strength development of cementitious materials [15,16]. Toda et al. [13] have shown that the strength of the mixtures was not determined by the bulk content of humic acids. Furthermore, that quantification of humic acids may not indicate the effect of soil organic matters on the strength of the mixtures. Since soil organic matter has an indefinite macromolecular structure with variety in elemental compositions and functional groups [17,18,19,20], specific components in the soil organic matters could affect the strength of the mixtures that was not quantified by the evaluation of bulk humic acid content. In addition, in the previous studies, the effects of specific components of soil organic matters on the strength development of the mixtures are not comprehensively understood.

The objective of this study is to determine whether particulars such as the elemental composition of humic acid in dredged soils can be used as a parameter that indicates dredged soils that form soft mixtures after mixing with a steel slag, in addition to known factors that work as indicators such as amorphous silica and portlandite content in dredged soils and steel slag, respectively. In addition, the results of this study could underpin whether soil organic matters in dredged soils does or does not affect the strength development of the mixtures. Tests of unconfined compressive strength of the mixtures made with eight dredged soils collected from eight different Japanese harbors and a steel slag are carried out with characterization of the humic acids of the dredged soils to compare the characteristics of hard and soft mixtures. The characterization of mineralogical phase compositions and solution compositions of the mixtures are carried out to address the possibility of how soil organic matters may affect the strength development of the mixtures.

## 2. Materials and Methods

### 2.1. Materials

#### 2.1.1. Dredged Soils

Eight dredged soil samples from different Japanese harbors were studied. Four dredged soil samples named soils A, B, C, and D also employed in Toda et al. [13] were used for further study of their soil organic matters. Four other dredged soil samples named soils E, F, G, and H were newly collected from different Japanese harbors for this study. The characterization of soils A, B, C, and D and analyses of their mixtures with steel slag were carried out in Toda et al. [13]. The results of characterization of soils A, B, C, and D and analyses of their mixtures was also reported in Toda et al. [13]. The characterization of soils E, F, G, and H and subsequent formation of mixtures followed the methodology of Toda et al. [13] to compare the characteristics of eight soil organic matters and strength development of the eight mixtures.

The physical properties of the dredged soils are summarized in Table 1. The soil particle density, liquid limit, and the content of fine particles were obtained following Japanese Industrial Standards A 1202, A 1205, and A 1204, respectively. The accuracy of soil particle density and liquid limit measurements were all in the range of ±0.01 g/cm^3^ and ±0.1%. The content of fine particles was obtained using sufficient amounts of sample, and the data accuracy typically is in the range of ±5%. All dredged soils were clay and silt rich, with common soil particle densities of soils (2.55 to 2.75 g/cm^3^) except soil G, which was slightly less dense than the other soils likely caused by soil organic matters [21].

Liquid limits of the dredged soils A, B, C, and D were 73.4, 89.8, 44.1, and 66.2% [13], respectively. Liquid limits of the dredged soil E, F, G, and H were 80.0, 54.5, 96.1, and 66.1%, respectively, which the liquid limit was used to adjust the water content of the dredged soils prior to mixing with steel slag.

**Figure 1:**
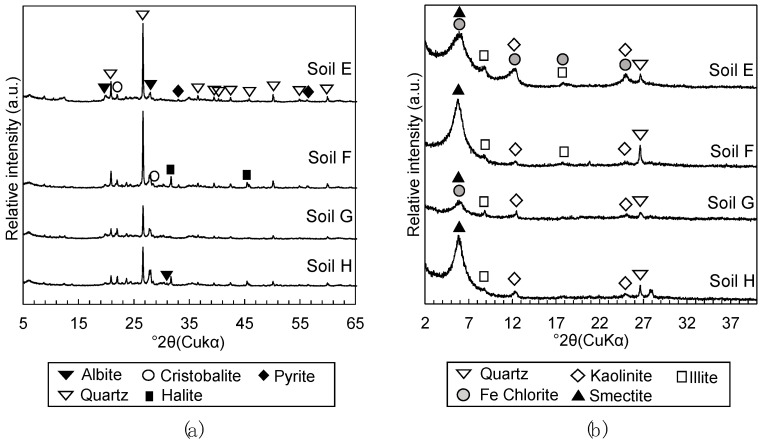
X-ray diffraction (XRD) patterns of (**a**) powdered dredged soil to detect bulk mineralogical phases and (**b**) oriented particles of dredged soils to detect clay mineralogical phases. Names of dredged soil samples are labeled on the upper right of each XRD pattern.

**Figure 2:**
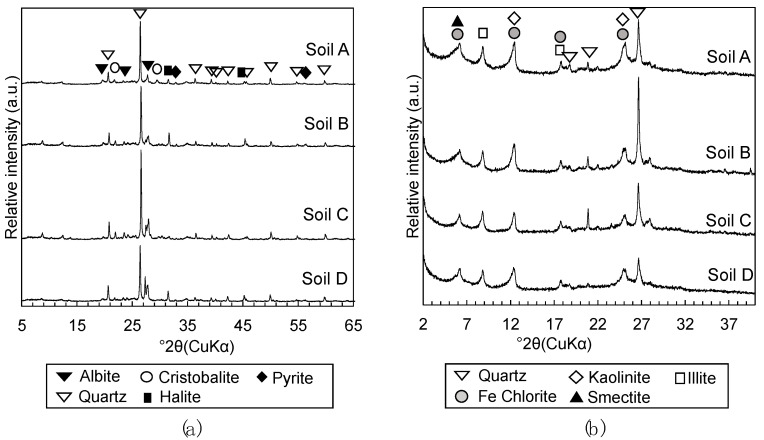
XRD patterns of (**a**) powdered dredged soil to detect bulk mineralogical phases and (**b**) oriented particles of dredged soils to detect clay mineralogical phases. Names of dredged soil samples are labeled on the upper right of each XRD pattern. From Toda et al. [13].

The characterization of soils E, F, G, and H followed the methodology described in Toda et al. [13]. The bulk mineralogy and clay mineralogy of the dredged soils were characterized with powdered samples pulverized to finer than 53 μm in diameter and oriented samples of particles below 2 µm in diameter prepared by elutriation by X-ray diffraction analysis (XRD; RINT2100, Rigaku, Tokyo, Japan). Inorganic amorphous silica contents were quantified by selective dissolution methods, where diatom frustules were dissolved by heating 50 mg of dried soil per 40 mL of 2M Na_2_CO_3_ at 85 °C for 5 h [22] and volcanic glass were dissolved by heating 50 mg of dried soil per 50 mL of 0.5 M NaOH at 100 °C for 2.5 min [23]. Dissolution experiments were repeated three times to calculate the average and standard errors of the results. Subsequently, the silica concentration of supernatants was measured by inductively coupled plasma-atomic emission spectroscopy (ICP-AES; ICPE-9000, Shimadzu, Kyoto, Japan) to quantify inorganic amorphous silica contents in the dredged soils. The bulk and clay mineralogy of soils E, F, G, and H (Figure 1) were virtually identical, and they were also very similar to soils A, B, C, and D [13] (Figure 2), containing quartz, cristobalite, albite, pyrite, kaolinite, illite, chlorite, and smectite. The total inorganic amorphous silica contents of soils E, F, G, and H, which were calculated as the sum of quantified diatom frustules and volcanic glass, were 79.6 ± 4.2, 64.3 ± 1.7, 90.0 ± 2.2, and 75.3 ± 3.1 mg/g respectively, while the inorganic amorphous silica content of soils A, B, C, and D reported in Toda et al. [13] were 78.2 ± 5.9, 71.4 ± 2.8, 44.4 ± 3.4, and 38.2 ± 1.5 mg/g, respectively.

#### 2.1.2. Steel Slag

**Figure 3:**
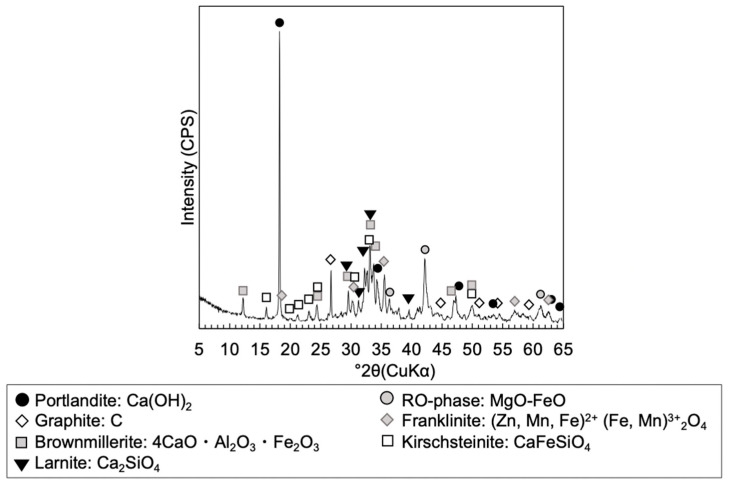
XRD pattern of powdered steel slag 1 showing mineralogical phases. From Toda et al. [13].

Steel slag 1, employed in Toda et al. [13], was used to form the steel slag-dredged soil mixtures in this study. Its mineralogical composition was confirmed to be similar to those of ordinary steel slag, containing larnite, brownmillerite, RO phase, calcite, and portlandite with other oxide phases, based on the XRD analysis (Multiflex Diffractometer, Rigaku, Tokyo, Japan) of powdered samples pulverized to finer than 53 μm in diameter [13] (Figure 3).

### 2.2. Methods

**Figure 4:**
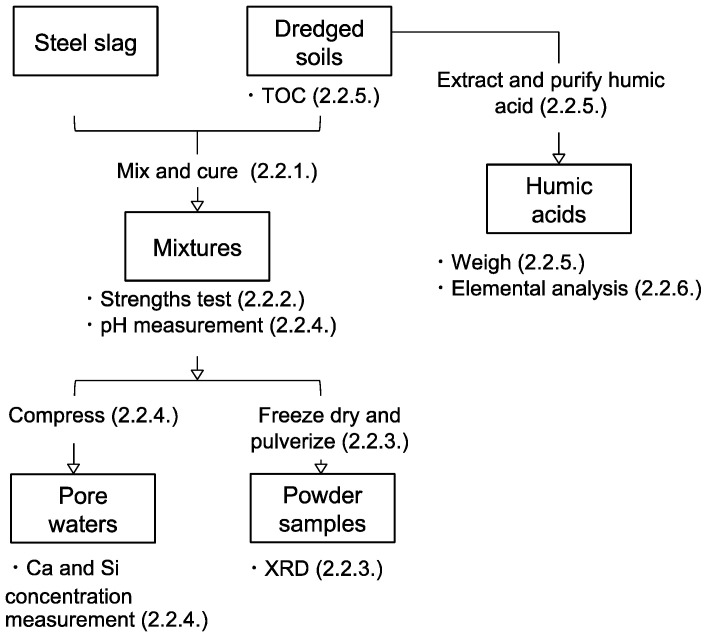
Diagram of experimental procedures.

A schematic diagram of the experimental methodology is shown in Figure 4. The framed words are the samples, and below the frames are the measurements conducted for each state of samples. Procedures for sample preparations are described between the connecting lines of the frames. Numbers in brackets refer to subsections of methods that describe each methodology.

#### 2.2.1. Preparation of Steel Slag-Dredged Soil Mixtures

The steel slag-dredged soil mixture specimens were prepared with steel slag 1 and soils E, F, G, and H by the method described in Toda et al. [13]. The mixtures of steel slag 1 and soils E, F, G, and H were termed mixtures 1E, 1F, 1G, and 1H, respectively. They were prepared by mixing steel slag into dredged soil in the volume ratio 3:7. Air-dried steel slag was used to make the mixtures. The water content of the dredged soils was conditioned to 1.5 times the liquid limit using artificial sea water to equalize the physical properties before mixing. The mixing was performed by an electronic mixer for 5 min; then, the mixture was packed into cylindrical plastic molds, of height of 100 mm and diameter of 50 mm, and covered with plastic film. Then, it was cured to conduct the subsequent tests of unconfined compressive strength. The mixtures were cured in a sealed plastic container in saturated humidity at 25 °C for the duration of the tests to eliminate reduction in the water content.

#### 2.2.2. Unconfined Compressive Strength Tests

The unconfined compressive strength (q_u_) was measured for mixtures 1E, 1F, 1G, and 1H cured for 3, 7, 14, and 28 days after demolding from the cylindrical plastic molds. The unconfined compressive strength tests were performed in triplicate for each condition and the average values and standard errors were calculated. The q_u_ values of mixtures made with steel slag 1 and soils A, B, C, and D were cured for 3, 7, 14, and 28 days were from Toda et al. [13]; these mixtures were termed 1A, 1B, 1C and 1D, respectively.

#### 2.2.3. Mineralogical Phases Assemblage

The XRD analysis (Multiflex Diffractometer, Rigaku, Tokyo, Japan) of mixtures 1E, 1F, 1G, and 1H for 1, 3, 7, and 28 days of curing were carried out with powdered samples. About 5 g of freeze-dried mixture was used for preparing powdered sample to reduce the effect of heterogeneity in the mixtures on the evaluation of the mineralogical compositions. Samples were pulverized to the grain size below 53 μm. The XRD patterns of mixtures 1A, 1B, 1C, and 1D for 0, 3, 7, and 28 days of curing were from Toda et al. [13]. The peak assignment was selectively carried out for phases that play a role in the pozzolanic reaction.

#### 2.2.4. Chemistry of the Pore Water Solutions

The pH of the pore water in mixtures 1E, 1F, 1G, and 1H cured from 1 to 28 days were measured directly from the mixtures using a pH meter employing a pH probe (1053B, Hanna Instruments, Woonsocket, RI, USA) with an electrode for semi-solids and soils. The pH measurements were performed three times, and average values and standard errors were calculated. The calcium and silica concentrations of the pore waters of the 1E, 1F, 1G, and 1H mixtures cured from 1 to 28 days and mixtures 1A, 1B, 1C, and 1D cured from 1 to 7 days were measured with pore waters collected by compressing one entire cylinder of the mixture specimens prepared by the plastic mold used for the unconfined compressive strength tests. One pore water sample was prepared for each condition, due to limitations in the amount of mixture sample. The collected pore waters were filtered by 0.2 μm membrane filters; then, calcium and silica concentrations were measured using inductively coupled plasma-atomic emission spectroscopy (ICP-AES; ICPE-9000, Shimadzu, Kyoto, Japan). The pH data of mixtures 1A, 1B, 1C, and 1D and calcium concentration data of mixtures 1A, 1B, 1C, and 1D from the 3-day cured samples were measured in Toda et al. [13] by the same method, which is shown in this study.

#### 2.2.5. Quantification of Soil Organic Matter and Humic Acids in the Dredged Soils

The total organic carbon (TOC) content in the dredged soils A, B, C, D, E, F, G, and H were measured in a TOC analyzer (TOC-L, Shimadzu, Kyoto, Japan) with 0.4 to 0.5 g of freeze-dried soils. The TOC content was calculated by subtracting the total inorganic carbon (TIC) content from the total carbon (TC) content. The measurements of TIC and TC were conducted twice, and average values and standard errors were calculated. 

**Figure 5:**
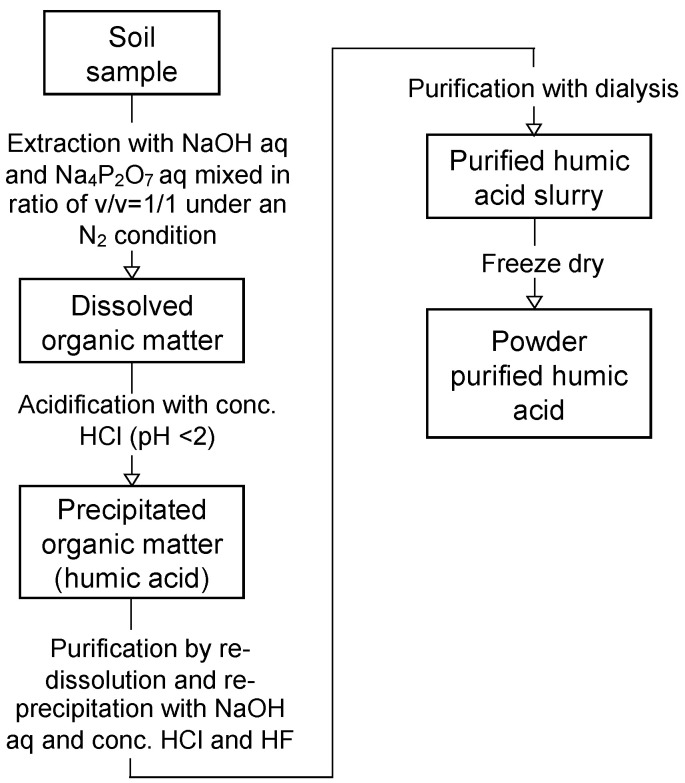
Diagram of humic acid extraction and purification procedures from the dredged soils.

The humic acids of soils E, F, G, and H were extracted from the dredged soils to quantify the humic acid content following the methodology in Fukushima et al. [24,25], which was the same to the humic acid extraction from soils A, B, C, and D in Toda et al. [13]. The humic acid extraction was not repeated, assuming the dredged soils to contain humic acid homogenously from the result of TOC measurement repetition. A schematic diagram of the humic acid extraction and purification method is shown on Figure 5. A 10 g of freeze-dried dredged soil and 100 mL of aqueous alkaline solution (1.0 M NaOH and 0.1 M Na_4_P_2_O_7_ mixed in volume ratio 1:1) were mixed and shaken under an N_2_ atmosphere for 24 h to solubilize soil organic matter. The condition of the extraction procedure is known to affect the recovery ratio of humic acids, and it must be kept the same between each soil samples to make comparisons of their humic acid content. Solubilized soil organic matter was collected after centrifugation of the mixture at 10,000 rpm for 15 min, and the supernatant was filtered using an A5 filter. Then, the filtrate was acidified to pH 1 by concentrated HCl (approximately 1 mL), which was stirred for 24 h. Then, the resulting precipitate was collected by centrifugation at 10,000 rpm for 15 min and filtration with a 0.20 μm filter. The precipitate was the soil organic matter that classifies to humic acid. Then, the purification of humic acid was carried out to remove coexistent inorganic phases. The re-dissolution of humic acid in 100 mL of aqueous 0.1 M NaOH was followed by the addition of concentrated HCl (1 mL) and HF (3 mL) to re-precipitate the humic acid. The slurry was stirred for 24 h and then centrifuged at 10,000 rpm for 15 min to collect the precipitate. Subsequently, the precipitate was dialyzed to remove coexisting ions such as Na. The precipitate was transferred to a dialysis tube (SpectraPore, nominal molecular weight cut-off of 1 kDa) and dialyzed against water for two weeks. The dialyzed slurry was freeze-dried in order to obtain a powder sample, which was weighed to quantify the humic acid content in the dredged soils. The weighing accuracy of the precision balance was ±0.0001 g, which gave the error range of ±0.001% in the calculation of humic acid content in the dredged soils.

The difference in TOC and humic acid content of common land soils and dredged soil samples could depict the characteristics of dredged soil samples. Hence, the TOC and humic acid content of standard land soils of the Japan Humic Substance Society (JHSS), Inogashira (Andosols) and Dando (Brown Forest Soils) soils, which are the two soil types that cover 61% of Japanese land [26], were used for the comparison of the soil organic matter contents [27].

#### 2.2.6. Elemental Compositions of Humic Acids

Analyses of C, H, N, and S contents of purified humic acid powder of soils A, B, C, D, E, F, G, and H were carried out at the Center for Instrumental Analysis at Hokkaido University. Prior to the analyses, the powdered humic acids were dehydrated under reduced pressure for at least 24 h. The C, H, and N contents were measured by an elemental analyzer (CE440, Exeter Analyzer, Warwickshire, UK). About 2 mg of sample was used for the measurement and WO_3_ was added as a combustion aid for the C, H, and N analysis. The maximum permissive error of measurement of C, H, and N contents was ±0.3%. The S contents were measured by an ion chromatography (Dionex ICS-1600, Thermo Fischer Scientific, Waltham, MA, USA). About 2 mg of sample was used for a measurement. The maximum permissive error of measurement of S content was ±0.3%. The content of oxygen was calculated by subtracting the sum of the quantified percentage of C, H, N, and S from 100%. The elemental compositions of JHSS standards [28], Dando, and Inogashira humic acids were referred in the results to make a comparison between humic acids of common land soils and dredged soil samples.

## 3. Results

### 3.1. Characterization of the Mixtures

#### 3.1.1. Unconfined Compressive Strength

**Figure 6:**
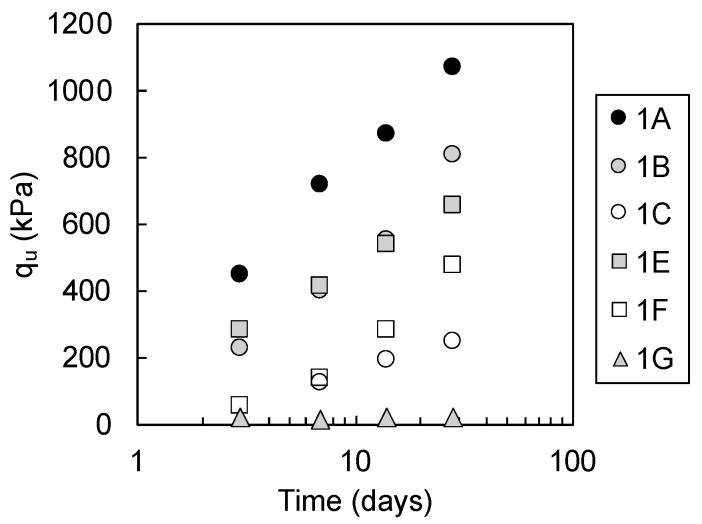
Unconfined compressive strength (q_u_) values of mixtures 1A, 1B, 1C, 1E, 1F, and 1G with curing time (data for mixtures 1A, 1B, and 1C are from Toda et al. [13]).

The average q_u_ values of the mixtures is shown in Figure 6. Error bars are omitted, as they typically fell within the range of the plot size, and they do not affect the interpretation on coordinates of the plots on the figure. Mixtures 1D and 1H did not develop sufficient strength to undergo unconfined compressive strength tests and the plots are not presented in Figure 6. The q_u_ value of mixture 1G was low throughout the 28 days of the curing period (18 to 28 kPa). Mixtures 1D, 1H, and 1F were classified as soft mixtures, which did not qualify for use as construction works with lower strength than the target strength of 250 kPa at 28 days of curing, calculated from required strength of 100 kPa with safety factor [29]. Mixtures 1A, 1B, 1C, 1E, and 1F with q_u_ values above 250 kPa at 28 days of curing were classified as hard mixtures, making them acceptable for use in construction work. Mixtures 1A, 1B, 1C, 1E, and 1F developed strength (increases in q_u_ value) during the curing period; mixture 1A showed the highest strength at 28 days of curing, followed by mixtures 1B, 1E, 1F, and 1C.

#### 3.1.2. Mineralogical Phase Assemblages

**Figure 7:**
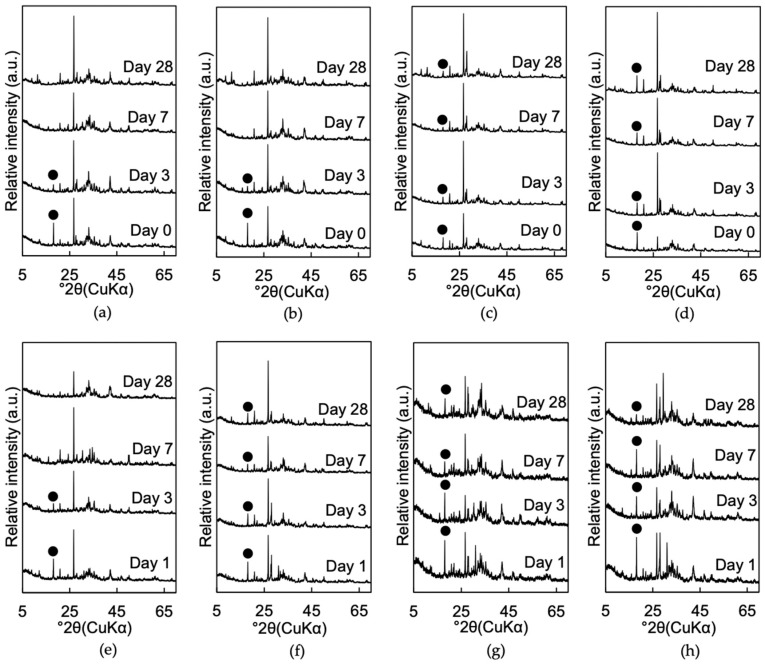
XRD patterns of (**a**) mixtures 1A, (**b**) 1B, (**c**) 1C, (**d**) 1D, (**e**) 1E, (**f**) 1F, (**g**) 1G, and (**h**) 1H cured for 1, 3, 7, and 28 days. XRD patterns (**a**–**d**) are from Toda et al. [13]. Black dot (●): Portlandite (Ca(OH)_2_).

The XRD patterns of mixtures 1A, 1B, 1C, 1D, 1E, 1F, 1G, and 1H are shown in Figure 7. The XRD patterns of mixtures 1A, 1B, 1C, and 1D are from Toda et al. [13]. The consumption of portlandite was the major change in the mineralogical phase assemblage in some mixtures as a function of curing time. Mixture 1E showed the consumption of portlandite after 7 days of curing with disappearance of the peak intensity corresponding to portlandite (Figure 7e). This tendency of the portlandite peak disappearance in mixtures exhibiting a q_u_ value above 600 kPa at 28 days of curing were also observed in mixtures 1A and 1B (Figure 7a,b). In mixtures 1F, 1G, and 1H, the portlandite still remained after 28 days of curing (Figure 7f,g,h) as did mixtures 1C and 1D (Figure 7c,d). Amorphous silica and C-S-H were not detected with XRD, due to their amorphous and poorly crystalline characteristics [14,30].

#### 3.1.3. Solution Chemistry of Pore Water

**Figure 8:**
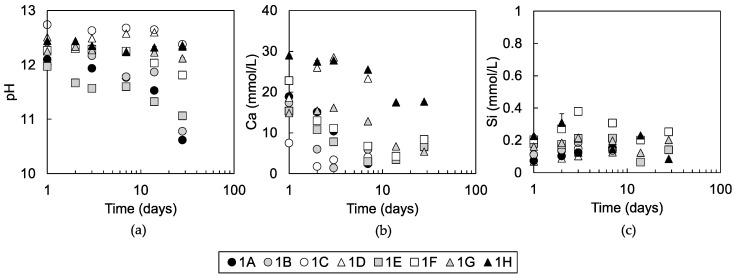
(**a**) pH of pore waters of mixtures 1A, 1B, 1C, 1D, 1E, 1F, 1G, and 1H at 1 to 28 days of curing, (**b**) calcium concentrations, and (**c**) silica concentrations of the pore waters of mixtures 1A, 1B, 1C, and 1D of 1 to 7 days of curing and of mixtures 1E, 1F, 1G, and 1H of 1 to 28 days of curing are plotted as a function of curing time. Standard errors of the measurements are plotted on the figures.

Figure 8 shows the solution chemistry of the pore water collected from the mixtures. Standard errors of pH, Ca, and Si measurements were mostly smaller than the size of the plots in the figures. In addition to data collected in this study, pH data of mixtures 1A, 1B, 1C, and 1D and calcium concentration data of mixtures 1A, 1B, 1C, and 1D at 3 days of curing time are cited from Toda et al. [13] and plotted in Figure 8a,b, respectively. The pH of pore water of mixtures 1E and 1F decreased from the order of 12 to lower pH values throughout the 28 days of curing, which was similar to mixtures 1A and 1B (Figure 8a). Mixtures 1G and 1H maintained pH around 12, which was similarly observed in mixtures 1C and 1D. The calcium concentration of mixtures 1D and 1H were around 30 mmol/L at least until 7 days of curing (Figure 8b). The calcium concentration of the pore water of mixtures 1A, 1B, 1E, 1F, and 1G gradually decreased from over 15 to below 10 mmol/L with curing time. Calcium concentrations of pore water of mixture 1C between 1 and 7 days of curing were maintained at around 5 mmol/L. Silica concentrations of pore waters of all mixtures were between 0.07 and 0.38 mmol/L during 28 days of curing (Figure 8c).

### 3.2. Characterization of Soil Organic Matter in Dredged Soils

The TOC and humic acid content of the dredged soils and JHSS standard soils are shown in Table 2 with elemental compositions of the corresponding humic acids. The TOC contents of the dredged soil samples were lower than Inogashira and Dando soils. The humic acid contents in the dredged soils were lower than those in the JHSS standards, except for soil G. The TOC and humic acid contents of soils A, B, C, D, E, F, G, and H varied from 0.60 ± 0.01% to 3.86 ± 0.01% and 0.09% to 0.93%, respectively. Soil G had higher TOC and humic acid content than that of the other dredged soils, as could be expected from it having the lightest soil particle density among the dredged soils (Table 1).

Humic acids in the dredged soils were mostly nitrogen-poor and sulfur-rich in comparison with the humic acids of JHSS standards. Inogashira humic acid contained 4.01% of nitrogen and 0.26% of sulfur, while Dando humic acid contained 4.49% of nitrogen and 0.29% of sulfur. Among humic acids of dredged soils, humic acids of soils D and H, with 7.23% and 7.04% of sulfur, respectively, contained much more sulfur than the other humic acids. Humic acids of soils D, G, and H were not as poor in nitrogen content as other humic acids of dredged soils in comparison with JHSS standards; they contained 3.25%, 3.93%, and 3.92% of nitrogen, respectively.

## 4. Discussion

### 4.1. Indicators for Strength Development of the Steel Slag-Dredged Soil Mixtures

**Figure 9:**
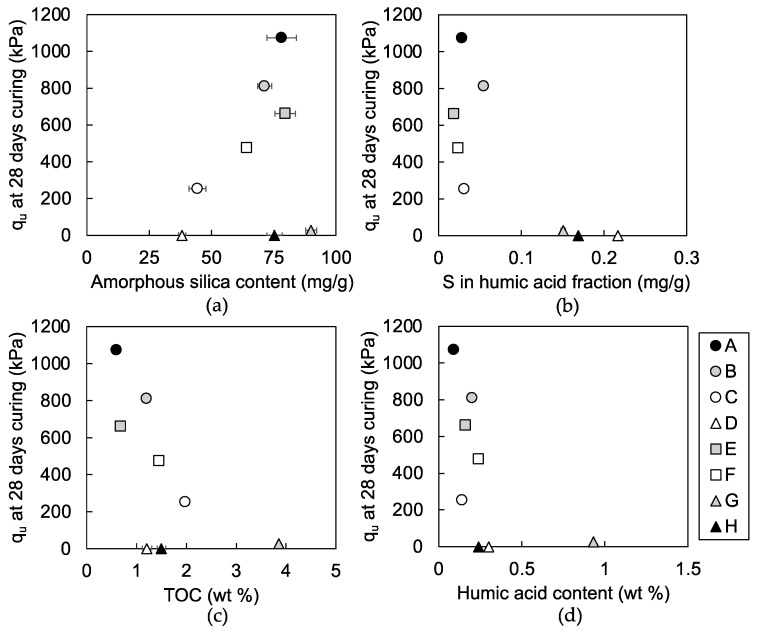
Relationship of (**a**) amorphous silica, (**b**) sulfur in the humic acid fraction, (**c**) TOC and (**d**) humic acid content per gram of dried dredged soil with q_u_ values of each steel slag-dredged soil mixtures at 28 days of curing. The q_u_ values for mixtures 1D and 1H are plotted as 0 kPa in order to include their plots in the diagram; they did not undergo unconfined compressive strength tests. The amorphous silica content of soils A, B, C, and D and the q_u_ values of mixtures 1A, 1B, 1C, and 1D are cited from Toda et al. [13]. Error bars of figure (**a**,**c**) show standard errors of data collected from repeated experiments and error bars of figure (**b**,**d**) show accuracy of the measurements.

Inorganic amorphous silica content in the dredged soils and portlandite content in steel slag have been pointed out as important factors for the strength development of steel slag-dredged soil mixtures [13]. Amorphous silica supplies dissolved silica and portlandite supplies dissolved calcium and establish alkaline condition for C-S-H formation via the pozzolanic reaction. The amorphous silica content of dredged soils was linearly correlated to q_u_ values of the mixtures, i.e., at 28 days of curing, except in soils G and H (Figure 9a). Soils G and H were two of the dredged soils classified as resulting in soft mixtures, with remaining portlandite after curing (Figure 7g,h), regardless of the similar content of amorphous silica to the soils, which formed hard mixtures (Figure 9a) with portlandite consumption (soils A, B, and E) (Figure 7a,b,e). This suggests that soils G and H contain components that may inhibit the pozzolanic reaction.

The sulfur content in the humic acid fraction distinguished soils G and H, and also soil D from soils A, B, C, E, and F (Figure 9b). Soils G, H, and D contained over 0.151 mg of sulfur per gram of soil in the humic acid fraction (Figure 9b), whereas soils A, B, C, E, and F contained sulfur below 0.05 mg/g. Therefore, the sulfur in humic acid fraction could suggest the occurrence of inhibition in the pozzolanic reaction and the subsequent strength development of the mixtures. Inhibition in the pozzolanic reaction of mixtures 1G and 1H was suggested to result from the effect of soil organic matters because soils G and H were rich in sulfur in the humic acid fraction and had sufficient dissolved silica supply similar to soils that formed hard mixtures: soil A, B, and E. Furthermore, inhibition in the pozzolanic reaction of the mixture 1D may result from the effect of soil organic matters, together with the limitation of dissolved silica supply because soil D was rich in sulfur in the humic acid fraction but had minimal amorphous silica content among the studied dredged soil samples.

In addition, as expected from Toda et al. [13], the TOC (Figure 9c) or humic acid content (Figure 9d) showed no relationship with q_u_ values of the mixtures, which emphasized the importance of interpreting the content of specific components of soil organic matters for the indication of dredged soils, which form soft mixtures.

### 4.2. Characteristics of Humic Acids that Inhibit Strength Development of the Mixtures

From the results in Figure 9b, it is clear that the inhibition of the strength development of the 1G, 1H, and 1D mixtures were indicated by the content of sulfur-bearing components in the humic acids. These may be directly attributed to the inhibition in the pozzolanic reaction of the mixtures, as well as there may be unquantified components in the soil organic matters that correlate positively to the sulfur-bearing components and may contribute to the inhibition of the pozzolanic reaction.

Intrinsic characteristics of humic acids of the soils G, H, and D were further investigated with a comparison of their elemental ratios, which have been used to evaluate the average properties, sources, and alterations of humic acids [19,31,32]. 

**Figure 10:**
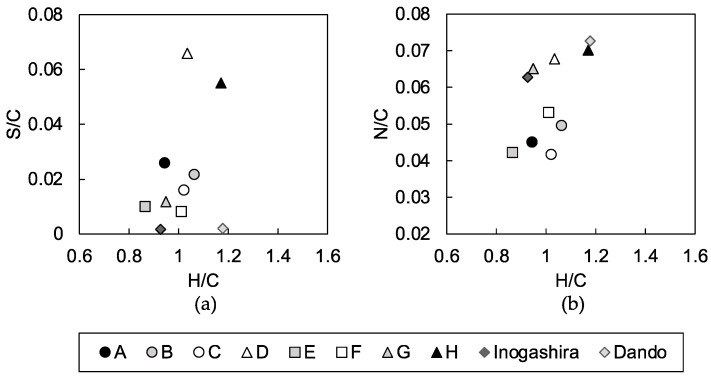
(**a**) S/C versus H/C atomic ratios and (**b**) N/C versus H/C atomic ratios of humic acids extracted from the dredged soils (A, B, C, D, E, F, G, and H), Dando humic acid and Inogashira humic acid.

The S/C of humic acids in all of the studied dredged soils was higher than that of the JHSS standards (Figure 10a). This may result from differences in the following: (1) the inclusions of sulfur-bearing organic compounds; (2) degree of abiotic reaction of reduced sulfur with organic matter [33], which increases the preservation of sulfur in sedimentary soils [34]; and/or (3) the redox state of the sedimentary soils [31] where reducing conditions are favorable to conserve sulfur in the sediments. Factors affected S/C of humic acids of dredged soils is not conclusive, though it could be further discussed with analysis of sulfur redox states of humic acids, which may indicate the redox condition of sedimentary soils. In addition, if sulfur-bearing components play a role in the inhibition of the pozzolanic reaction, their redox state would be critical for a full understanding of the inhibition mechanism of the pozzolanic reaction. The speciation of sulfur in humic acids of sedimentary soils is commonly assumed to be disulfides, thiols, sulfonates, or ester-bonded sulfates [34,35]; however, these various chemical properties would act differently on the pozzolanic reaction.

Furthermore, Figure 10a shows that the S/C ratio of humic acid of soils D and H were almost twice as large as the S/C ratio of humic acids of other dredged soils and JHSS standards. The sulfur content in the humic acid fraction of dredged soils could indicate the inhibition of a pozzolanic reaction of the mixtures made with soils D and H (Figure 9b), because their humic acids had a high S/C ratio. The inhibition of pozzolanic reaction of the mixture made with soil G, whose humic acid had a similar S/C ratio to the humic acids of dredged soils that formed hard mixtures, was also indicated by the sulfur content in the humic acid fraction of dredged soils (Figure 9b) because the content of humic acid in soil G was high. Characteristics of humic acids are suggested to vary between dredged soils that form soft mixtures. The input of organic matters to the sedimentary soils and sulfur enrichment in the soil organic matter structure shape the sulfur content in the humic acid fraction of dredged soils as an indicator of dredged soils that form soft mixtures.

The nitrogen content in the humic acid fraction of the dredged soils did not clearly indicate dredged soils that form soft mixtures as sulfur did; hence, nitrogen-bearing functional groups are not likely a candidate of soil organic matters that inhibit the pozzolanic reaction. However, the N/C versus H/C plot suggested that the N/C ratio of the humic acid could be a characteristic of the dredged soils that form soft mixtures (Figure 10b). The humic acids of soils D, G, and H plot between the two JHSS standards of N/C of 0.062 to 0.073. The higher N/C of soil organic matter has been suggested to result from the sedimentation of marine organic matter rich in amides, and/or as a result of low aeration, which could conserve the compositions of the sedimented organic matter [19]. Stuermer et al. [31] supported the former factor when they observed that algal sources, rather than highland plant sources, caused N/C enrichment.

The causes of sulfur and nitrogen enrichment of humic acids in dredged soils are not determined due to several possible causes of the enrichment. Despite the requirement of further investigations to clarify specific components in soil organic matters that may contribute to the inhibition of the pozzolanic reaction, the discussion here highlights that: (1) the content of such organic components in dredged soils is suggested to be affected by the input of organic matters to sedimentary soils and the sedimentary environment; (2) the N/C of humic acids also indicate the dredged soils that form soft mixtures as the sulfur content in the humic acid fraction of dredged soils does. Overall, these newly discovered indicators of dredged soils that form soft mixtures when mixed with a steel slag, together with quantification of their inorganic amorphous silica content, would enhance the utilization of dredged soils by accelerating the evaluation processes of their strength development.

### 4.3. Effects of Soil Organic Matters on Pozzolanic Reaction

The factors that affect the pozzolanic reaction are calcium and silica sources, or other coexisting components such as soil organic matters that inhibit the reaction, as emphasized by mixtures 1D, 1G, and 1H. Soil organic matters in soils D, G, and H could interact with the pozzolanic reaction by pH buffers to the weak alkaline region [15]; the formation of calcium-organic matter complexation [36], and mineral surface coverage [37,38,39]. Such reactions would cause the solution chemistry to be undersaturated vis-a-vis C-S-H, which disables its precipitation, decreases calcium supply to C-S-H formation, and inhibits phase dissolution or precipitation, which could inhibit the pozzolanic reaction.

Among these possibilities, the effect of a pH buffer by soil organic matters was not significant in any of the mixtures as discussed in Toda et al. [13]. The pH of the pore water in the mixtures was above 12 after 1 day of curing in all mixtures (Figure 8a). From the comparisons of calcium concentrations of pore water in cement-treated soils of Tremblay et al. [15], which showed organic reagents to cause one to two order higher concentrations of calcium in solution with the control sample of Tremblay et al. [15] and our data, it is speculated that the formation of calcium complexation may not be significant in the mixtures 1D, 1G, and 1H. The occurrence of surface coverage by soil organic matters could not be clarified, yet it is clear that the dissolution of portlandite was not inhibited, as shown by the pH and calcium concentrations of pore water in mixtures 1D, 1G, and 1H at saturation of portlandite with pH around 12.5 and calcium concentration above 20 mmol/L (Figure 8a,b). In addition, silica concentrations of all mixtures at all curing times were similar (Figure 8c), so the soil organic matters in soils D, G, and H may not inhibit the dissolution of amorphous silica either, which leaves precipitation sites of C-S-H as possible surface-covering sites for soil organic matters. It was confirmed that a pH buffering effect does not play a role in the inhibition of the pozzolanic reaction in steel slag-dredged soil mixtures. The calcium complexation ability of soil organic matters in soils D, G, and H, and its ability to cover the mineral surface will be investigated to determine how pozzolanic reaction inhibition occurs in the mixtures made with soils D, G, and H in future research.

## 5. Conclusions

The inhibition of strength development in steel slag-dredged soil mixtures by soil organic matters is detailed. Our data especially highlight the importance of the quantification of specific components among soil organic matter in evaluating the effect on the strength development of such mixtures. Dredged soils with enriched sulfur content in the humic acid fraction, and/or N/C ratio of extracted humic acids similar to that of land humic acids, resulted in the formation of soft mixtures. These characteristics could be used as indicators of dredged soils that form soft mixtures, together with the inorganic amorphous silica content in the dredged soils. Also, the TOC and humic acid content in dredged soils are not suggested as indicators of soft mixture formation. Our findings clarify that the strength development indicators have significant implications for the utilization of steel slag-dredged soil mixtures. The discovery of indicators of strength development of steel slag-dredged soil mixtures will accelerate evaluation processes for the strength development of mixtures made with newly sampled dredged soils by making the estimation of their strength development possible, which would facilitate and promote the utilization of steel slags and dredged soils.

Subsequently, the inhibition of the pozzolanic reaction by soil organic matters that form soft mixtures was not due to the pH-buffering capacity of soil organic matters but may be occurring because of calcium complexation or mineral surface coverage by soil organic matters. An understanding of the inhibition mechanism of the strength development requires further studies, clarification of what component in the soil organic matters to attribute to the inhibition of the pozzolanic reaction, and how this component inhibits it; however, the results show the potential of this study for further investigations to facilitate the validation of dredged soils by mixing with cementing additives that form C-S-H as the major binding phase via the pozzolanic reaction. Overall, the findings should contribute to an increased utilized fraction of dredged soils and industrial by-products that act as alkaline activators that replaces the destination of such materials from disposal wastes to construction resources, which would promote their utilization in the construction industry.

## Figures and Tables

**Table 1:** Physical properties of dredged soils.

Samples	Soil Particle Density (g/cm^3^)	Liquid Limit	Content of Fine Particle (<0.075 mm) (%)
Soil A ^(1)^	2.777	73.4	83.2
Soil B ^(1)^	2.737	89.8	99.3
Soil C ^(1)^	2.709	44.1	91.1
Soil D ^(1)^	2.707	66.2	58.6
Soil E	2.721	80.0	75.5
Soil F	2.655	54.5	86.3
Soil G	2.544	96.1	99.3
Soil H	2.629	66.1	63.5

^(1)^ From Toda et al. [13].

**Table 2:** Total organic carbon (TOC), humic acid content and humic acid elemental compositions of soils A, B, C, D, E, F, G, and H. The values of Inogashira and Dando soils, Japan Humic Substance Society (JHSS) standards, are also listed for comparison.

Samples	TOC	Humic Acid Content	Humic Acid Elemental Composition (wt.%) ^(1)^				
	(wt. %)	(wt. %)	C	H	N	S	O ^(3)^
Soil A	0.60 ± 0.01	0.09 ^(2)^	45.55	3.61	2.39	3.14	45.31
Soil B	1.20 ± 0.01	0.20 ^(2)^	47.26	4.22	2.73	2.72	43.07
Soil C	1.97 ± 0.01	0.14 ^(2)^	52.03	4.46	2.53	2.20	38.78
Soil D	1.21 ± 0.10	0.30 ^(2)^	41.12	3.57	3.25	7.23	44.83
Soil E	0.68 ± 0.01	0.16	43.33	3.15	2.13	1.14	50.25
Soil F	1.46 ± 0.04	0.24	46.17	3.92	2.86	0.99	46.06
Soil G	3.86 ± 0.01	0.93	51.76	4.12	3.93	1.62	38.57
Soil H	1.50 ± 0.08	0.24	47.88	4.70	3.92	7.04	36.46
Inogashira ^(4)^	16.7	5.10	54.83	4.27	4.01	0.26	36.63
Dando ^(4)^	6.19	0.69	53.04	5.25	4.49	0.29	36.93

^(1)^ Maximum permissive error of elemental analysis was ±0.3%. ^(2)^ From Toda et al. [13]. ^(3)^ Calculated by subtraction from 100%. ^(4)^ TOC and humic acid content from Kuwatsuka et al. [27] and elemental composition from Watanabe et al. [28].
